# Medicine and Its Practitioners during the Earlier Years of the History of Bath
^1^The Presidential Address at the meeting of the Bath and Bristol Branch of the British Medical Association, delivered at the Annual Meeting held in Bath, May 28th, 1913.


**Published:** 1913-09

**Authors:** James Wigmore


The Bristol

Medico-Chirurgical Journal.

*"Scire est nescire, nisi id me
Scire alius sciret."*

SEPTEMBER, 1913.

### MEDICINE AND ITS PRACTITIONERS DURING THE EARLIER YEARS OF THE HISTORY OF BATH.[1]

BY

JAMES WIGMORE, M.D.

IN thinking over a subject on which to address you, I found considerable difficulty in deciding. My desire was to choose a subject not too polemical, as I look upon these meetings more as the scientific side of our Society. There are occasions enough when we can thrash out what I might call the political or business work of the branch; I therefore refrain from referring to the Insurance Act and its working, which might be supposed to have a good deal of interest for members at the present time.

I shall ask your indulgence while I place before you some disjointed notes on medicine and its practitioners in Bath, as

[1] The Presidential Address at the meeting of the Bath and Bristol Branch of the British Medical Association, delivered at the Annua Meeting held in Bath, May 28th, 1913.

far as I have been able to gather, during the early years of its history. Agreeing with Mr. Balfour, as I do, that " to make the best use of the future one must never ignore the past," and as George Augustus Sala wrote, " There is to me an inexpressible charm in the lives of the good, brave, learned men whose only objects have been to alleviate pain and to save life," or as Emerson said, " Nature seems to exist for the excellent. The world is upheld by the veracity of good men. They make the earth wholesome. Other men are the lenses through which we read our own minds."

I thought it might not be amiss to consider how our predecessors in medicine bore themselves in their day. We are the heirs of their labours, and it becomes us to consider how we uphold and use their inheritance. Let us therefore " praise men of renown and our fathers in their generation." " And there are some of whom there is no memorial, who are perished as if they had never been."

Now, in looking up the lives of former Bath physicians one is struck by the fact that the men whose names have come down to us as prominent in the life of the city were all connected with the practice of the Bath waters. There may be one or two exceptions, but speaking generally, Bath physicians have distinguished themselves by their writings in connection with the waters.

Prebendary Scarth, in his history, says, referring to ancient Bath physicians, " It is most probable that a School of Medicine existed in Bath at an early period. The mineral springs being visited by many patients for their healing benefits would naturally cause the residence of eminent physicians in the neighbourhood." He shows, however, that there is no record of any of these or of their patients ; neither is there any account of a votive altar put up by a physician, as, for instance, at Chester ; neither has any memorial to a physician been discovered such as has been found on the line of the Roman Wall in Northumberland. The only thing to show that medicine was practised here during the Roman occupation was a medicine stamp, which was dug up in the Abbey Churchyard in 1731.

A Mr. Mitchell, of Bristol, had it in his possession for some time, but there is at present no trace of it. This stamp probably belonged to an empiric. It was a stone of greenish hue, perforated, and of oblong form. The inscriptions on it have been translated, from which it would appear he was an oculist. They are as follows :—

1st side. The thalasser of Titus Junianus for clearing eyesight.

2nd ,, The crysomelinum of T. Junianus for clearing eyesight.

3rd ,, The diexum or dryxum of T. Junianus for removal of old scars.

4th ,, The phœbum (or blistering collyrium) of T. Junianus for such hopeless cases as have been badly treated or given up by the physicians.

Scarth explains that the dryxum was probably compounded from gall nuts and used as an astringent.

Other medicine stamps have been found at Cirencester and Uttoxeter, and Dr. McCaul remarks that medicine stamps present more difficulty to the antiquary than any other Roman remains.

The reverend author, however, shows that the Roman armies which visited Britain were provided with medical officers, for he writes : " Notices of physicians who attended those Roman Emperors who visited Britain have been preserved. Thus, Scribonius Largus is stated to have accompanied Claudius, and Severus was attended by his own physicians." It would appear the emperor's son " Caracella attempted to induce his father's medical attendants to hasten the emperor's death," but for the honour of the profession failed in this. However, he took his revenge, for when he became emperor himself he caused the physicians to be put to death for not complying with his wishes. Truly a poor reward for doing one's duty.

The Romans held possession of Bath for four hundred years, and during that time it was greatly improved by them, and was a place of great resort for its waters. We have still our Roman baths to show and bear witness to this. After the

Romans left and during the Saxon dominion (577–1066) there seems to be no reference to medicine or its practitioners in Bath; still, we may assume there were "leeches" here as elsewhere in England, whose practice consisted of a mixture of magic and medicine; but when religious houses were founded the care of the sick was undertaken by their inmates, though there were no separate or individual practitioners. Every ecclesiastic was more or less a physician, and secular canons were trained in nursing. A recent writer says, "This much is certain, that Christianity, in so far as it consisted in tending the weak and suffering, in so far as it meant the formation of humane, gentle and beneficent purposes by organised combinations, was realised most fully in those early monastic retreats."

It may be interesting to add, as Bath was closely identified with the Benedictines, this community was the first religious body to study the science of medicine and to aim at a higher standard. We do not find among the Anglo-Saxons (according to Dr. Payne) any mention of high ecclesiastics as themselves leeches or of leeches reaching high ecclesiastical rank, such as we come across later among the Anglo-Normans.

The coming of the Normans, though it did not assist the progress of the science of medicine in England, as for nearly a century after the Conquest there is no trace of any Anglo-Norman medical literature, nevertheless made a difference in the social position of the medical profession. It is plain that the Norman aristocracy required physicians of their own nationality, as in the first place they did not understand or speak English, and secondly, they would probably despise the old Anglo-Saxon leeches. Besides, the Norman doctors were educated abroad, most of them at Salerno, and there was no school of medicine in England at the time; and so we find the first physician of whom we have an account in Bath was John De Villula, or as he was generally called John of Tours. He was appointed to the See of Somerset, which was then located at Wells (1088). The late Dr. Tunstale maintained the name was John de Pillula, in playful allusion to his earlier profession. Authorities differ as to whether he practised medicine in Bath. Warner, in his history of Bath, says he was "an empirick, who amassed a fortune by imposing on the ignorance and credulity of the invalids who flocked to the healing waters of this city in search of ease and health," and that being anxious to safeguard his possessions, he became an ecclesiastic.

The late Austin King says John was at once chaplain and physician to William Rufus. He had amassed a fortune by the practice of physic, and was attracted to Bath by what he had heard of the medicinal fame of its waters; but other authorities do not consider he practised medicine here. Be that as it may, he proved himself a true friend of Bath; for after being appointed to the See of Somerset, which was then located at Wells, he removed the See from Wells to Bath. Bath, which had been burnt and sacked by Bishop Geoffrey, sallying from Bristol, he restored. He obtained from the king a grant of the whole of the city of Bath—the church, the abbey, the mint, the baths, rights, customs, tolls, etc., in pure and perpetual alms for the augmentation of the See of Somerset, and for the special end that he might set up in that place his episcopal chair. He rebuilt the city, erected a cathedral which for grandeur and beauty was only equalled by the later structures of Wells and Gloucester. The baths he restored or constructed; one called the Bishop's Bath and the other the Prior's Bath were for the free use of the public. So that he was justly called the second founder of Bath.

The beauty of the baths after De Villula restored them is spoken of by the author of a work called *Gesta Stephani*, written in 1138. "There is," says this writer, "a city distant six miles from Bristol, where through hidden channels are thrown up streamlets of water warmed without human agency, and from the very bowels of the earth, into a receptacle beautifully constructed with chambered arches. These form baths in the middle of the city, warm and wholesome, and charming to the eye. Sick persons from all England resort thither to bathe in these healing waters, and the strong also to see these wonderful burstings out of warm water and bathe in them."

It may be worth noting in passing that to this learned physician is due the fact that the See of Somerset is known to-day as Bath and Wells. William of Malmesbury says that Bishop John "thought it not at all honourable to live without fame in a mere village (Wells), and had it in his mind to transfer his throne to Bath." In 1075 the Council of London decreed that the episcopal sees should be transferred from villages or small towns to greater centres of population; but whatever his motive was, the canons of Wells were not resigned to the change.

The dispute was finally settled by Pope Innocent IV (1245), who ordered that the throne of the bishop should be in each of the churches of Bath and Wells; that the election should take place in each church in turn, first at Bath, next time in Wells; that the style of the bishop in charter and in seal should be henceforward "Bishop of Bath and Wells."

De Villula seems to have been rather severe with the monks when he was made bishop, "because they were stupid, and in his opinion mere barbarians, and he took away all the lands upon which they depended for food, and doled out by his lay stewards a scanty allowance. However, after a time, by the introduction of foreign monks, and the effect of his overlooking, he became reconciled, for in 1106 he made over again to the monastery what he had taken away. In a grant, after stating how he laboured, and at last duly effected with all decent authority that the head and mother church of the Bishopric of Somerset shall be in the city of Bath in the church of St. Peter, he says: "I have restored to them (the monks) the lands which I formerly held unjustly in my possession." His repentance is described as geographical, for though he made amends in Bath he made none in Wells.

He is described as a skilled physician, and a man of learning and refinement and of social tastes. In his life he was simple and fearless, in his death he was resigned, and he provided that whatsoever he had begun in life should be completed after his death. He died in 1122, after thirty-four years' pontificate, and was buried before the altar in his cathedral church of St. Peter, Bath.

There is a long interval before we meet with the name of another Bath physician. A writer in the *Practitioner* of December, 1905, suggests that John Phreas, or Free, a learned physician born in London at the end of the fourteenth or beginning of the fifteenth century might be considered a Bath doctor. Phreas was made Bishop of Bath and Wells, but he does not seem to have practised in Bath ; indeed, he practised medicine only on the continent, and was bishop for only a month, as he died in Rome before taking up his residence at Wells. In a list of Bishops of Bath and Wells, given in Professor Earl's book (*Bath, Ancient and Modern*) his name is not included.

Perhaps I might just mention that Thomas Linacre, founder and first President of the College of Physicians, was made Dean of Wells after he took orders, and it requires no great stretch of imagination to think that he might have been interested in the baths.

We can gather what the condition of medicine about this time was in England from Linacre's biographer. He states : "The practice of medicine was scarcely elevated above that of the mechanical arts, nor were the majority of its practitioners better educated than mechanics. No society as yet existed independent of the monastic and ecclesiastical which could at all be considered learned." (The bishops or their vice-generals were the persons who could grant licences to practice medicine, in addition to the universities.) This was no doubt true as a general rule, though Linacre himself is an example of the very opposite. The age of scholasticism was being replaced by humanism. Linacre was described as a "medical humanist," and such medical men as could travel abroad, especially to Italy, to complete their education, were certainly among the best *literati*, so that in the fifteenth and sixteenth centuries medicine was closely allied to literature ; and one characteristic of our early medical humanists was their altruistic tendency. Linacre, who founded the College of Physicians as well as Lectureships at Oxford, and Caius who wrote the epitaph of Linacre, and founded a college at Cambridge, are lasting testimonies to this trait.

Still, we must not suppose there were no practitioners amongst the people, as in 1543 an Act was passed for protection and toleration of those irregular practitioners who kept shops and who were called apothecaries; and it would seem that some of these at any rate attained to some eminence, as Henry VIII, even after the grant to the College of Physicians, appointed an apothecary to the Princess Mary. Indeed, it was not until after 1815, when an Act was passed which gave the Apothecaries' Society power to prosecute persons who practised without the licence—this did not, of course, interfere with anyone holding a surgeon's or physician's qualification—that medical men generally can be said to have been educated, as previous to this their education was entirely optional, and a great many were illiterate and uneducated. So it is not to be wondered at that few names have come down to us in Bath until the sixteenth century.

Before the dissolution of the monasteries the monks were under obligation to keep the King's Bath and some houses suitable for royal lodgings near it in proper order for the occasional use of the king, and in Henry III's reign (1235) a process was issued calling upon the monks to pay £13 11s. 0d. (equal to £300 of our money) for their repair. It was common practice up to the middle of the fifteenth century for males and females to bathe together *in puris naturalibus*, and Bishop Beckyngton in 1449 ordered that the distinguishing garments of breeches and petticoats be worn on these occasions. But it was not until the middle of the sixteenth century, after the baths had been granted to the Mayor and Corporation by Elizabeth, that a stop could be put to this practice.

Connecting the practice of the monks with the change after the Dissolution, we meet with the name of Abbot Feckenham. During Mary's reign he was appointed Abbot of Westminster, but was deprived of this when Elizabeth came to the throne. He seems to have obliged Elizabeth in some way while she was princess, and when she came to the throne she tried to induce him to accept the new order; but he refused, and suffered twenty-three years' imprisonment. He was sometimes released on parole. It was during one of these intervals he resided in Bath, and being moved with compassion to see how the poor were excluded from the use and benefit of the medicinal waters, he built with his own means a small bath and hospital. It was furnished with seven beds for the most miserable objects who came to Bath for relief from her waters. It was at the corner of Nowhere Lane, and so near "The Leper's Bath" that the poor had little difficulty in stepping from one place to the other. He wrote a book with the heading, "This booke of sovereigne medicines against the most common and known diseases both of men and women was, by good proofe and longe experience, collected by Mr. Doctor Ficknam, late Abbot of Westminster (1576), and that chieflie for the poor which hath not att all tymes the learned phisitions att hande." In Freeman's book is a note that Dr. Caius, who succeeded Linacre as President of the College of Physicians, wrote a book (which was never published) called "De Thermis Britannicis," in which he gave a prominent place to the Bath waters, and the writer concludes from this that he must have practised at Bath for a time, probably after his return from Padua. He certainly practised at Cambridge, Norwich and Shrewsbury, and it is therefore possible that he was for a time at Bath.

I must confess to a feeling of satisfaction in the thought that Caius, who was the companion and fellow-lodger of Vesalius (justly named the Father of Modern Anatomy) at Padua and Aristotelian lecturer in conjunction with Realdus Columbus (also a great anatomist, who, if he did not discover, at any rate was the first to describe in writing the circulation through the lungs) was with some probability a Bath practitioner. As you are aware, Caius returned from Padua with the intention of introducing dissecting into England, and became one of the founders of Gonville and Caius College, Cambridge.

The next writer on the Bath waters is William Turner, D.D. He is frequently described as a Bath physician, but Freeman says he never practised at Bath, and indeed did not even know of the waters until he was Dean of Wells. Still, this does not preclude his practising at Bath, as he continued to practise medicine during his whole life. I quote Woods' description of him: "William Turner, M.D., D.D., Dean of Wells, was born at Morpeth, in Northumberland, educated at Cambridge in trivials and afterwards for a time in the study of medicine. This person, who was very conceited of his own worth, hot headed, a busybody, and much addicted to the opinon of Luther, would needs in the height of his study of physics turn theologist, but always refused the usual ceremonies to be observed in order to his being made priest. . . . Sure it is that while he was a young man he went, unsent for, through many parts of the nation and preached the word of God, not only in towns and villages, but also in cities."

There is no record that he took a degree in Arts and Medicine. After being imprisoned, he travelled in Italy, and was made Doctor of Physic at Ferrara, where he was held in much esteem for his faculty. He returned to England under Edward VI, and was made Prebendary of York, Canon of Windsor, and Dean of Wells. He was "incorporated Doctor of Physic of Oxford," and practised amongst the nobility and gentry, and became physician to Edward Duke of Somerset. He also, as was the case with many laymen who were scholars, obtained a licence to read and preach. He left England when Mary ascended the throne, and went to Germany, and from there to Rome, and afterwards settled for a time at Basel. About this time he published a book on birds, "little in size but great in value." He was also a botanist, as Pulteney, referring to his book on herbs, says "the true era of botany in England must commence with Dr. William Turner, who was unquestionably the earliest writer among us that discovered learning and critical judgment in the knowledge of plants, and whose book of herbs will always grow green and never wither as long as Dioscorides is held in mind by us mortal wights." He returned to England under Elizabeth, and was much thought of for his two faculties and the great benefits he did by them. He became a member of the House of Commons, and he claims to be the first writer on the Bath waters in his book entitled *A Book of the Nature and Properties of Baths in England and of other Baths*

*in Germany and Italy.* The preface is addressed to his "well-beloved neighbours of Bath and Bristol, Wellis Wymans and Chard," and is dated from Basel, March, 1557. Fox says of him: "In scientific investigation he was patient, wise and imperturbable." In the theological controversies of the day he was "ignorant, contentious, querulous, lacking indeed all the great qualities needed in a religious reformer." We, however, have only to deal with the man of science, and in that capacity we recognise in Turner a man of genius, the calm and patient investigator, and to all intents and purposes the father of Bath water literatures. It may not be out of place to give one or two extracts from the book of this Father of English Physic, as he was called, as it tells us how they took the baths in Elizabeth's reign.

Turner writes: "Now for the manner of bathing. I will not set down what the *Physician* is to do, but leave that to his judgment and discretion, but what is fit for the patient to know; for there are many cautions and observations in the use of bathing drawn from the particular constitutions of bodies, from the complications of diseases, and from many other circumstances which cannot be comprehended in general only or applied to all bodies alike. But many times upon the success and the appearing of accidents the Physician must *ex re nata capere consilium*' and perhaps alter his intended course, and perhaps change the bath to a hotter or cooler, etc. In which respect those patients are ill-advised which will venture without their Physician upon any particular bath or to direct themselves in the use of it, and this is a great cause that many go away from hence without benefit, and then they are apt to complain *of our baths* and blaspheme the great blessing of God bestowed upon us." I should imagine there could be little doubt that he practised at Bath after this quotation. He gives directions for a patient when he enters a bath: "It is well to have the head covered from the air and wind and from the vapours arising from the bath, also the kidneys, if they be subject to the stone, anointed with some cooling Unguents as Rosatum comitissae, Infrigidans Galeni, Santolinum, etc. Also to begin quietly with the bath till his body be inured to it, and to be quiet from swimming or much motion which may offend the head by sending up vapours thither." At his coming forth to have his body well dried and to rest in his bed an hour and sweat, etc. He says, " The morning hour after the sun hath been up an hour or two is fittest for bathing, and if it be thought fit to use it again in the afternoon it is best four or five hours after a light dinner." One wonders how a patient at the present day would manage to get his bath. " For the time of staying in the bath it must be according to the quality of the bath and the toleration of the patient. In a hot bath an hour or less may be sufficient ; in a temperate bath two hours. For the times of continuing the baths there can be no certain time set down, but it must be according as the patient finds amendment. Somtimes twenty days, sometimes thirty, and in difficult cases much longer, and, he says, " therefore they reason without their host which assign themselves a certain time as perhaps their occasions of business will best afford." " For the time of the year our Italian and Spanish authors prefer the spring and fall, and so they may well do in their hot countries ; but with us, considering our climate is colder and our baths are for cold diseases, I hold the warmest months in the year to be best, as May, June, July and August," and he adds, " I have persuaded many hereunto who have found the benefits of it." He says, " The variableness of our climate, and that people finding it temperate at noon, may be deceived as to the coldness of the mornings and evenings, and so offend weak constitutions." " Likewise in the bath itself, although the springs arise as hot as at other times, yet the wind and air beating upon them do them much harm and also make the surface of the water much cooler than the bottom." Therefore " it were to be wished that our Queen's Bath and Cross Bath, being small baths, were covered, and their slips made close and warm." " By this means our baths would be useful all the year, when neither wind and cold air in winter nor the sun in summer should hinder our bathing." And he adds, " I desire not novelties nor to bring in innovations, but I propound these things upon good grounds and examples of the best baths in Europe, and so I desire to have them considered of, referring both this point and whatsoever I have said in this discourse to the censure of those who are able to judge." "I do purposely omit many things about the virtues and uses of our baths, which belong properly to the Physitian, and cannot well be intimated to the patient without dangerous mistaking, for as Galen saith, 'Our art of Physick goes upon two legs—reason and experience—and if either of these be defective our Physick must needs be lame. *Experience* was first in order.

> "'Per Varios usus artem experientia fecit
> Exemplo monstrante viam.'
> "'From much experience the art of Physick came
> Directed by example to the same.'

Reason followed, which without experience makes a mere contemplative and theoretical Physitian. Experience without reason makes a mere Emperick, no better than a nurse or an attendant upon sick persons, who is not able, out of all the experience he hath, to gather rules for the cure of others. Wherefore they must be both joined together, and therefore I refer Physitian's work unto Physitians themselves.'"

Dr. Turner refers frequently to the Physitians practising here, and that there were a good many about this time may also be gathered from Harrison's description of Bath, 1577–78, published in Holinshed's *Chronicles*. After describing Bath in Somersetshire ("where I parson Harrison have been") and discussing the origin of its name, showing "the citie itself is a verie ancient thing no doubt as may yet appear by diverse notable antiquities ingraved in stone to be seene in the wals thereof,"[1] he discusses the origin of the heat of the waters. He says, "Dr. Turner, also the Father of English Physick and an excellent divine, supposeth that these springs doo draw their forces from Sulphur or if there be ani other thing mingled withall he guesseth that it should be Saltpetre because he found an obscure likelihood of the same even in the Crosse Bath." "But that they participated with anie Allume at all he could

[1] The stone had a Latin inscription, now defaced, others with inscriptions that can be read, also an inscription on a tomb.

never till his dieing daie be induced to beleeve." Harrison says Bath is very pleasantly situated and well supplied with water, and has four gates, bridges, etc. And describing the baths, he mentions the hot bath as being "very worthilie so called." "For at first coming into it men thinke that it would scald their flesh and lose it from the bone, but after a season and after that the bodies of the Commers there-to be warmed thoroughlie in the same it is more tolerable and easier to be borne." He says, "Hot houses in some countries are no better than brothels," and describing the King's bath, says, "It is compassed about with a verie high stone wall and the brims thereof are mured round about wherein lie two and thirtie arches for men and women to stand in separatlie, who being of the gentre for the most part, doo resort thither indifferentlie, but not in such lascivious sort as unto other baths and hot houses of the maine, whereof some write more a great deal than modestie should reveale and honeste performe." He speaks of the colour of the waters, and says, "At high noone and midnight all entrance into them is utterly prohibited, for at those two seasons and awhile before and after they boil and become so hot that no man is able to indure their heat or anie while sustain their force and vehement working." He then explains that on this account and "because of the filth which the diseased do leave in them, and so as to avoid any one catching a new disease from bathing, the baths are shut up from half an hour after ten o'clock in the forenoon to half an hour after one o'clock in the afternoon and likewise at midnight at which times the keeper of them resorteth to his charge, opening the gates and leaveth, or should leave, free passage unto such as come unto them." He continues: "Much money has been lately spent on the Baths and that they are better ordered, cleanlier kept, and more friendlie provision made for such povertie as dailie repaireth thither. But notwithstanding all this, such is the general estate of things in Bath that the rich men maie spend while they will and the poore beg while they list for their maintenance and diet, so long as they remaine there: and yet I denie not but that there is very good order in that Citie for all." "But where shall a man find anie and equal regard of poore and riche though God dooth give these his good gifts freelie unto both alike. I would here intreat further of the customs used in these baths what number of Physicians dailie attend upon those waters, for no man (especially such as be able to entertain them) dooth enter unto these baths before he consult with the Physician."

With the exception of Dr. Turner and Dr. John Jones, the names of these numerous physicians are buried with them.

Jones was a Welshman, educated at Cambridge and Oxford, but principally at Cambridge, where he took his degree in medicine, and practised in Bath and sometimes in Nottinghamshire and Derbyshire. In 1572 he published his book on the Bath waters, its title was *The Bathes of Bathes Ayde Wonderful and most excellent against very many sicknesses. Approved by Authoritie. Confirmed by reason and dayly tryed by experience. With the antiquitie commoditie, propertie, Knowledge, use, aphorismes, diet, Medicine, and other things thereto to be considered and observed."* The late Professor Earle fell foul of the title of the book as follows: "Dr. Jones was a Welshman, and he seems to have bungled in the English name of his book, which is quoted sometimes as the *Bathe of Bathes Ayde*, and sometimes as *The Bathes of Bathes Ayde*, of which title the grammatical construction is not readily apparent." Jones might, indeed, be said to be the first author of Bath water literature, as he devoted a whole book to the subject.

It was a characteristic of the times for writers to dedicate their books to some powerful and eminent patron, and so we find Dr. Jones following the custom in dedicating his book to "The Right Honourable Henry, Earle of Pembroke, Lord Herbert of Kayderdid," etc. An extract or two from the dedication may not be uninteresting. He writes: "Democritus the most Ancient Philosopher of Abdera, Reader to the Prince of Phisicyans, Hippocrates, (right honourable Earle) most learnedly in his Epistle de Natura Humana, to the same Hippocrates recounteth how necessary it is for all men to know the arte of Phisike. Bicause it is not onlie an understanding most honourable and profitable to lyfe. But also for that of all other it most manifestly setteth forth to the Sences the wisedome power providence and unmeasureable bountie of our almightie Creatour of which to be ignorant, it is great impitie, as Galen testifieth in his Thyrd Booke, 'De usu Partium.'" He goes on to explain "that of all sortes Phisike is to bee embraced, and of them cheefely which are endued with honourable dignities and waightie affaires of the Commonwealth." "For," he continues, "as wysedome (sister to Phisicke) dooth deliver the myndė from evill affectes and maketh us to live forever in perpetuall joye with Aungelles. So Phisike maintayneth health and expelleth sicknesses from the body making us live a long and lustie lyfe, as Galen in his workes (De Sanitate tuenda) most reasonablie teacheth." After explaining how necessary it is to have health in order to enjoy life, he tells my Good Lord how "all men ought to take care of their health as unless the body is sound the mind itself is affected and how sickness darkneth the mynde, dulleth the sences, and depriveth, deminisheth or depraveth the partes."

It would seem that even in Jones's time there were not wanting many critics ready to have their fling at an author, for he tells the "Noble and Prudent Earle" that "while sparing no pains nor fearing the reproachful words of the envious Momous and his capacious rabble," he published "An Ayde most profitable for all them that need it . . . seeing that among all the most marvellous works of nature there is none more wonderful, none more excellent, none more available to the help of the diseased . . . than the Baths natural of the Citie of Bath if they be rightly used and orderly observed, and as need requireth frequented."

He divided his book into four parts, or rather, as he himself says, "he included four books in one volume." The first book treats of the descent of Bladud, etc.; the second of the different opinions concerning the cause of the Hot Waters, what minerals are in them, and what are the qualities by which they work their effects; the third treats of the diseases for which the Waters are useful and things against nature, with all the signs of the state of the sick and whole, through which the better Consultation may be had not only whether these Bathes will help or not, but also the Chyrurgians students in Physicke and all other capable of reason may fynde a most apte trade of understanding, comprehended in few words. The fourth and last book gives " Aporisms and brief rules how in and at the Bathes they shall use themselves, what meates and drinkes what cordiall comfortatines with most excellent purgations, clisters suppositoures etc. meet for every complection and purging humours, abounding with all other remedies against such accidents as grow by reason of hot Bathes and to what infirmitie every of the Bathes serve beste severally." He lauds his patron very profusely, ending by humbly beseeching "the Almighty to endue your good Lordship and the right honourable Lady Anne, your noble and moste virtuouse wife with Galens health, Nestors yeares, Crœsus welth and Augustus hapiness." Some of his "Aporisms" are rather quaint. The first runs as follows: "Acknowledge yourselves with the Holy Apostle Paul to be in the Lord's hands as the pot is in the potters, saying before you go in altogether on your knees devoutly the prayer appointed in the end of the book." (This is a special prayer composed by Jones. It commences by acknowledging that the diseased state of our body is due to our sins and wickedness.) He advises that anyone with a fever, anyone whose temperature is hot and dry, children springalles, young men lean, consumed, also women great with child must eschew the baths, and that all persons affected or grieved by a journey should rest a day or two before entering the baths.

Our author quotes " Agricola " as saying " that one should not take the baths in the pestilent season nor in the full of the moon according to Rolandus, nor in a leap year sayeth Savonarola, because it is the year of Saturn." But he will not agree with this last advice, as he says the leap year is simply means invented to make the year even, and cannot therefore have anything to do with Saturn. He advises taking the bath in the months of April, May, June, September and October, when the air is temperate, as being the best times. About an hour after sunrise in the morning is the time to have a bath, and if the disease require it *drink the Water out of the Spring.* "So much of the Water as shall not be grievous to the stomach may be drunk." "Every person entering shall first emptie his bellie and make water and if so be he cannot do that every day yet every second or third day." He advises a bath morning and evening if the state of the body and strength shall require. He says, "The fat, strong, cold, moist and women do sustain longer tarriance in the bath, that is two hours in the morning and one and a half hours in the evening. In the Bath you should neither eat nor drink neither by the space of an hour after coming forth except necessitie contravening." He advises after coming out of the bath to cover yourself with clothes, go to bed, and sweat. Sleep after sweating, he says, is convenient, but avoid sleeping while in the bath. If you be weak or have the joint ache take exercise after dressing and use "fricacion of the outward parts." If fricacion (which here shall be rubbing with nettel clothes) shall not be made, we must use a suppositour of honie or of the root of white lettuce or lard or soap. He gives directions for the diet, saying it ought to be of light digestion and nourishment, not gross, not over cold, nor vehemently hot. He says bodies of birds and four-footed beasts are better nourishment than fish, and these both than fruits or herbes. He recommends that the meat and fish be boiled, not roasted nor fried, or powdered either with spices or salt. He forbids quails, pigeons, sparrows, or such hot, filling meats. Fruits, as almonds, raisins, prunes and quinces baked or any way dressed, shall not be permitted. He says, "If in the first day the bellie by the bath be shrunken together toward the backbone it is a good and wholesome sign. But if the wombe be puffed up or affected with ache or else onwhile it is hot and another while cold it is an evil sign." It is worthy of note that in Jones's book we find mention for the first time of drinking the waters, as this very subject gave rise to some heat in a discussion between Guidott and Pierce. Years after Jones's time Guidott claimed to be the first to recommend it, and Dr. Pierce pointed out that Jones ordered it more than a hundred years before. But that practitioners were rather shy of ordering patients to drink the waters for long after Jones's time is obvious from a letter of Sir Thomas Browne to Dr. Pierce (written in 1677), in which he recommends one Mrs. Bridget Reave, of Suffolk, to his care, and proposed her drinking the Waters as well as bathing for a chlorosis cachexia, and in which he added these words "If my old friend Dr. Bave [another Bath physician] had taken more notice of my counsel the drinking of the Bath Waters might have been in use long ago: for above thirty years since I writ unto him to bring the drinking of them into use according to the custom of many other Baths beyond sea, which he very well knew, but would not hazard his credit in such a new attempt which notwithstanding had not been an innovation but rather a renovation, or renewing a former custom."

The list of doctors of the sixteenth century draws to a close with the name of Dr. Reuben Sherwood. Guidott says of him: "Of Dr. Reuben Sherwood, the first Physician I meet with any remembrance of, I can give no other account than that he died here Anno Domini 1598." Dr. Sherwood was born in Bath, and little is known of him except that he was the father of Dr. John Sherwood, who graduated in the University of Cambridge, and died in 1620, and was buried in the Abbey. The inscription on a brass against the wall over his grave is in Latin, but the translation as given by Guidott is—

"Here famous Dr. Sherwood lies,
Whose skill in Physick lore
Was great and his bright fame yet flies
Both now and evermore.

"Although within this tomb his bones
Are hidden out of sight
His soul not pent within these stones
To Heaven hath ta'en her flight."

He occupied Abbey House, where he received wealthy and illustrious patients until his death. Queen Anne during her visit to Bath stayed at Abbey House as Dr. John Sherwood's patient.

Dr. Thomas Elton was a contemporary of Dr. Sherwood, and was buried at Bath, August 11th, 1618. As Guidott says, "A well-bred gentleman, obliging and affable." He was a local man, and most probably educated *here*, as he does not seem to have graduated at either University.

I have now finished with the Bath physicians to the end of the sixteenth century, and I do not propose to proceed with my account any further. To refer to the many notable men of the seventeenth century would require an address to itself. As Dr. Parker in his Presidential Address in 1911 said, "It was a century of the greatest importance in the history of medicine on account of the brilliant development of natural science and medicine in England at that time." Besides, I fear I have already taxed your patience overmuch, and I will conclude with a quotation from one of the Bath writers of this seventeenth century, Dr. Pierce; and we may learn a lesson from these old worthies that a good name is more than wealth, and honest reputation better than great success, and that an amiable, upright member of his profession leaves a sweeter memory than a more learned mountebank. Pierce says, "But seeing that no calling is more disgraced than by the men of the same calling . . . I wish all Professors of Physick to carry themselves worthy of their calling, to be faithful and honest in their courses, not to insinuate with any after the manner of our Bath guides, presse upon them to be retained. If an Emperick or Mountebank seek about work, I blame them not, let them deceive those who will be deceived but for such as are graduated in the noble faculty of Physick to do so it is Fidler-like, a note if not some unworthinesse in them I am sure of a base mind. Let those therefore that are Physicians indeed strive to maintaine the reputation of their art and not by a base insinuating Carriage or Mountebanklike tricks to get a note and repute, vilifie their owne worth or disgrace so noble a faculty."

